# Major Adverse Events Associated With Emergency Endotracheal Intubation in Adult Patients: A Systematic Review

**DOI:** 10.7759/cureus.114540

**Published:** 2026-08-14

**Authors:** Laila Ahmed Abdelhamid, Ahmedelmostafa Amin Hussien Mahmoud, Lamyaa El Hassan, Irfanullah Khan, Abdulrahman Osman Derar, Sabah Saeed Hassan Dafaa Alla, Eman Ali Mohamed Hassan, Abdallah Adel Hassan Mohamed Abdelsalam

**Affiliations:** 1 Emergency Medicine, Dr. Faris Al Haddaj Hospital, Sakaka, SAU; 2 Anaesthesia, Saudi German Hospital, Ajman, ARE; 3 Basic Medical Sciences, College of Medicine, Jazan University, Jazan, SAU; 4 Accident and Emergency, Frimley Park Hospital, Frimley, GBR; 5 Emergency Medicine, Dr. Sulaiman Al Habib Hospital, Riyadh, SAU; 6 Emergency Medicine, Pilgrim Hospital Boston, United Lincolnshire Hospitals NHS Trust, Boston, GBR; 7 Paediatrics, University Hospitals Sussex NHS Foundation Trust, Sussex, GBR; 8 General Medicine and Gastroenterology, Withybush Hospital, Haverfordwest, GBR

**Keywords:** airway management, cardiovascular collapse, emergency endotracheal intubation, first-pass success, hypoxaemia, peri-intubation cardiac arrest, systematic review

## Abstract

Emergency endotracheal intubation (ETI) is among the highest-risk procedures performed in acutely ill adults, yet the burden, spectrum, and determinants of major peri-intubation adverse events remain unevenly characterised across settings. This systematic review synthesises contemporary primary evidence on major adverse events complicating emergency ETI in adults.

PubMed/MEDLINE, Embase, Scopus, Web of Science, and ClinicalTrials.gov were searched from January 2021 to July 2026 for peer-reviewed primary studies (randomised trials, prospective or retrospective cohorts, and case-control studies) reporting major adverse events after non-elective ETI in adults outside the operating theatre. Reviews, meta-analyses, protocols, abstracts, editorials, letters, and preprints were excluded. Data were extracted in duplicate; observational studies were appraised with the Newcastle-Ottawa Scale and randomised trials with the Cochrane RoB 2 tool. Findings were synthesised narratively in accordance with the Preferred Reporting Items for Systematic Reviews and Meta-Analyses (PRISMA) 2020.

Fourteen studies comprising approximately 61,430 intubation encounters across 14 countries were included. At least one major adverse event was reported in 14-52% of emergency intubations, with composite event rates of 45.2% in a 29-country cohort and 32.3% in a national emergency department cohort. Cardiovascular instability was consistently the dominant event (20.0-43.4%), followed by severe hypoxaemia (9.3-18.5%) and peri-intubation cardiac arrest (1.0-5.4%). Risk rose steeply with each additional laryngoscopy attempt (adjusted odds ratio: 4.4 for two attempts and 13.9 for four) and with pre-intubation hypotension, hypoxaemia, acidaemia, and elevated lactate. Major adverse events were independently associated with mortality.

Major adverse events complicate a substantial minority to majority of emergency intubations in adults and are driven principally by pre-existing physiological derangement and repeated attempts. Standardised outcome definitions and pre-intubation haemodynamic optimisation warrant priority attention.

## Introduction and background

Indications for emergency endotracheal intubation (ETI) range from acute respiratory failure and depressed consciousness to haemodynamic collapse, making it one of the most frequently performed high-acuity procedures across emergency departments, intensive care units, and general hospital wards. Unlike elective intubation in the controlled environment of the operating theatre, emergency ETI is performed in patients with limited physiological reserve, incomplete clinical information, and little opportunity for optimisation, often under significant time pressure and sometimes by less experienced operators. As a result, a procedure associated with a very low complication rate in elective anaesthetic practice can become a high-risk intervention in critically ill patients. Although ETI is frequently lifesaving, it is also associated with potentially severe complications, highlighting the importance of understanding and preventing adverse events during the peri-intubation period. First-pass success has therefore become a central quality indicator of emergency airway management, as each additional laryngoscopy attempt prolongs apnoea, increases the risk of oxygen desaturation, and may worsen haemodynamic instability [1]. Pooled data from emergency department series place benchmark first-pass success at approximately 84%, a threshold that has since been widely adopted for audit and quality improvement [2]. Contemporary difficult airway guidance accordingly emphasises structured preparation, preoxygenation, and explicit contingency planning rather than device selection alone [3].

The adverse events associated with emergency ETI are conventionally classified into major and minor categories. Major events, often referred to as major peri-intubation adverse events, include severe hypoxaemia, new or worsening cardiovascular instability, and peri-intubation cardiac arrest, and some definitions additionally include unrecognised oesophageal intubation, pulmonary aspiration, and death occurring within one hour of the procedure. Minor events include dental or lip trauma, mainstem bronchial intubation, and transient dysrhythmias. This distinction is clinically important because major events, unlike minor complications, represent potentially fatal complications occurring during a period when patients have minimal physiological capacity to tolerate additional stress. Hypoxaemia and hypotension during the peri-intubation window can compromise cerebral and myocardial perfusion and may contribute directly to poor outcomes. A synthesis of critically ill populations estimated that major adverse events occur in a substantial proportion of emergency intubations, although reported rates vary considerably depending on the clinical setting and definitions used [4]. Such variability represents an important challenge, as inconsistent thresholds for defining hypoxaemia, hypotension, and other complications limit comparisons between studies and hinder global benchmarking of emergency airway outcomes.

Significant efforts have focused on identifying modifiable factors associated with adverse outcomes during emergency ETI. Video laryngoscopy has been associated with improved first-pass success compared with augmented direct laryngoscopy in registry analyses [5] and with higher success rates among trauma patients after propensity matching [6], while operator experience and seniority remain consistent predictors of procedural success across different training environments [7]. More recent research has shifted attention from anatomical difficulty alone toward physiological vulnerability, with increasing evidence identifying pre-intubation shock, hypoxaemia, and metabolic acidosis as major contributors to peri-intubation cardiac arrest [8]. Despite these advances, available evidence remains fragmented across international cohorts, national registries, single-centre studies, and pragmatic clinical trials, with considerable variation in patient populations, clinical settings, and outcome definitions. A comprehensive synthesis of primary studies focusing specifically on emergency ETI in adults is therefore needed to clarify the burden of major adverse events and identify factors associated with their occurrence. The objectives of this systematic review were to determine the incidence and spectrum of major adverse events associated with emergency ETI in adults, identify patient-level and procedural determinants of these events, and evaluate their relationship with mortality.

## Review

Materials and methods

Study Design

This systematic review was conducted and reported in accordance with the Preferred Reporting Items for Systematic Reviews and Meta-Analyses (PRISMA) 2020 statement [9]. The review was not prospectively registered; the protocol, including the eligibility criteria, the search strategy, and the analytic plan, was specified in writing before screening commenced and was not amended thereafter. As the review used only published aggregate data, ethical approval and informed consent were not required.

Search Strategy

PubMed/MEDLINE, Embase, Scopus, Web of Science, and the ClinicalTrials.gov registry were searched from January 1, 2021, to July 15, 2026; the trial registry was searched to identify completed studies with published peer-reviewed results. The date restriction was applied a priori under the review protocol: a preliminary scoping search identified more than 10 eligible studies, and the search was therefore limited to the most recent five-year window so that the synthesis would reflect contemporary airway practice, including routine video laryngoscopy, apnoeic oxygenation, and post-pandemic infection-control workflows. The search combined controlled vocabulary and free-text terms across three concept blocks: (i) intubation terms ("intubation, intratracheal"[MeSH] OR endotracheal intubation OR tracheal intubation OR airway management OR rapid sequence intubation OR laryngoscopy); (ii) setting terms (emergency OR emergency department OR emergency service, hospital OR critically ill OR resuscitation OR non-elective); and (iii) outcome terms (adverse event* OR complication* OR peri-intubation OR hypoxaemia OR hypoxemia OR desaturation OR hypotension OR cardiovascular collapse OR haemodynamic instability OR cardiac arrest OR oesophageal intubation OR mortality). Blocks were combined with the Boolean operator AND. No language restriction was applied at the search stage. The reference lists of all included articles and of relevant recent syntheses were hand-searched to identify additional eligible reports. Detailed search strings are provided in the Appendices section.

Eligibility Criteria

Eligibility was framed using the Population, Intervention/Exposure, Comparator, Outcomes, and Study design (PICOS) structure, which permits the precise and reproducible specification of inclusion and exclusion criteria [10]; the full framework is presented in Table 1. Studies were eligible if they enrolled adults (predominantly aged 18 years or older) undergoing non-elective ETI performed outside the operating theatre and reported the incidence, determinants, or consequences of at least one major peri-intubation adverse event. Eligible designs were randomised controlled trials and observational primary studies, including prospective and retrospective cohorts, registry analyses, cross-sectional studies, and case-control studies. Studies were excluded if they were narrative or systematic reviews, meta-analyses, conference abstracts, editorials, letters, commentaries, study protocols, preprints, case reports, case series of fewer than 20 patients, simulation or manikin studies, or animal studies; if they were restricted to paediatric or neonatal populations; if they addressed elective operating-theatre intubation only; or if they reported first-pass success without any major adverse event outcome. Where multiple reports arose from the same parent cohort, each was retained only if it addressed a distinct research question and reported non-duplicative outcome data; such overlap is identified explicitly in the synthesis.

**Table 1:** Eligibility criteria based on the PICOS framework ED: emergency department; ICU: intensive care unit; PICOS: Population, Intervention/Exposure, Comparator, Outcomes, and Study design

Domain	Inclusion	Exclusion
Population	Adults (predominantly ≥18 years) undergoing non-elective endotracheal intubation	Neonatal or paediatric cohorts; mixed-age cohorts without adult-specific data
Intervention/Exposure	Emergency intubation outside the operating theatre (ED, ICU, or ward); any device, drug, or preoxygenation strategy	Elective operating-theatre intubation; supraglottic or surgical airway as the index procedure
Comparator	Any comparator (device, drug, subgroup, number of attempts, risk category); single-arm cohorts eligible if event rates were reported	None (absence of a comparator was not itself an exclusion)
Outcomes	≥1 major peri-intubation adverse event: severe hypoxaemia, cardiovascular instability or collapse, cardiac arrest, oesophageal intubation, aspiration, or peri-intubation death	First-pass success, glottic view, or intubation time reported without any major adverse event outcome
Study design	Randomised trials, prospective or retrospective cohorts, registry analyses, cross-sectional studies, case-control studies	Reviews, meta-analyses, abstracts, editorials, letters, protocols, preprints, case reports, series <20 patients, simulation, and animal studies
Other	Peer-reviewed, published January 1, 2021, to July 15, 2026; full text available; any language	Wholly duplicative outcome data from one cohort; full text unobtainable

Study Selection

Records retrieved from all sources were exported to EndNote X9 (Clarivate Analytics, Philadelphia, PA, USA) and deduplicated. Titles and abstracts were screened independently against the eligibility criteria, and the full texts of all potentially eligible records were then examined in duplicate. Disagreements were resolved by discussion and consensus, with recourse to a third assessor where necessary. Reasons for exclusion at the full-text stage were recorded and are reported in the PRISMA 2020 flow diagram [9].

Data Extraction

Data were extracted into a piloted standardised form and cross-checked. Extracted fields comprised the following: author, publication year, country and number of participating centres, study design and enrolment period, clinical setting, eligibility criteria, sample size, patient demographics, indication for intubation, intubation technique and device, exposure or intervention, comparator, definitions of each major adverse event, the incidence of each event, adjusted effect estimates for determinants of adverse events, and mortality. Where an article reported both crude and adjusted estimates, the fully adjusted estimate was extracted. No attempt was made to impute missing data or to contact study authors; unreported items are recorded as not reported.

Risk of Bias Assessment

Methodological quality was appraised independently in duplicate using design-appropriate validated instruments. Non-randomised studies were assessed with the Newcastle-Ottawa Scale (NOS), applying the cohort or case-control version as appropriate, across the domains of selection (maximum four stars), comparability (maximum two stars), and outcome or exposure ascertainment (maximum three stars) [11]; total scores of 7-9, 5-6, and 0-4 were classified as low, moderate, and high risk of bias, respectively. Randomised trials were assessed with the revised Cochrane Risk of Bias tool for randomised trials (RoB 2) across its five domains, namely, the randomisation process, deviations from intended interventions, missing outcome data, measurement of the outcome, and selection of the reported result, with an overall judgement of low risk, some concerns, or high risk [12]. Discrepancies were resolved by consensus.

Data Synthesis

Clinical and methodological heterogeneity across study designs, settings, and adverse event definitions precluded meaningful statistical pooling; accordingly, a quantitative meta-analysis was not undertaken, and results are synthesised narratively, structured by adverse event category, by determinants of harm, and by association with mortality. Incidences are reported as they appeared in the source articles, with the accompanying denominators and event definitions preserved. Because no meta-analysis was performed, formal assessment of publication bias by funnel plot or Egger's test was not applicable.

Results

Study Selection

**Figure 1:**
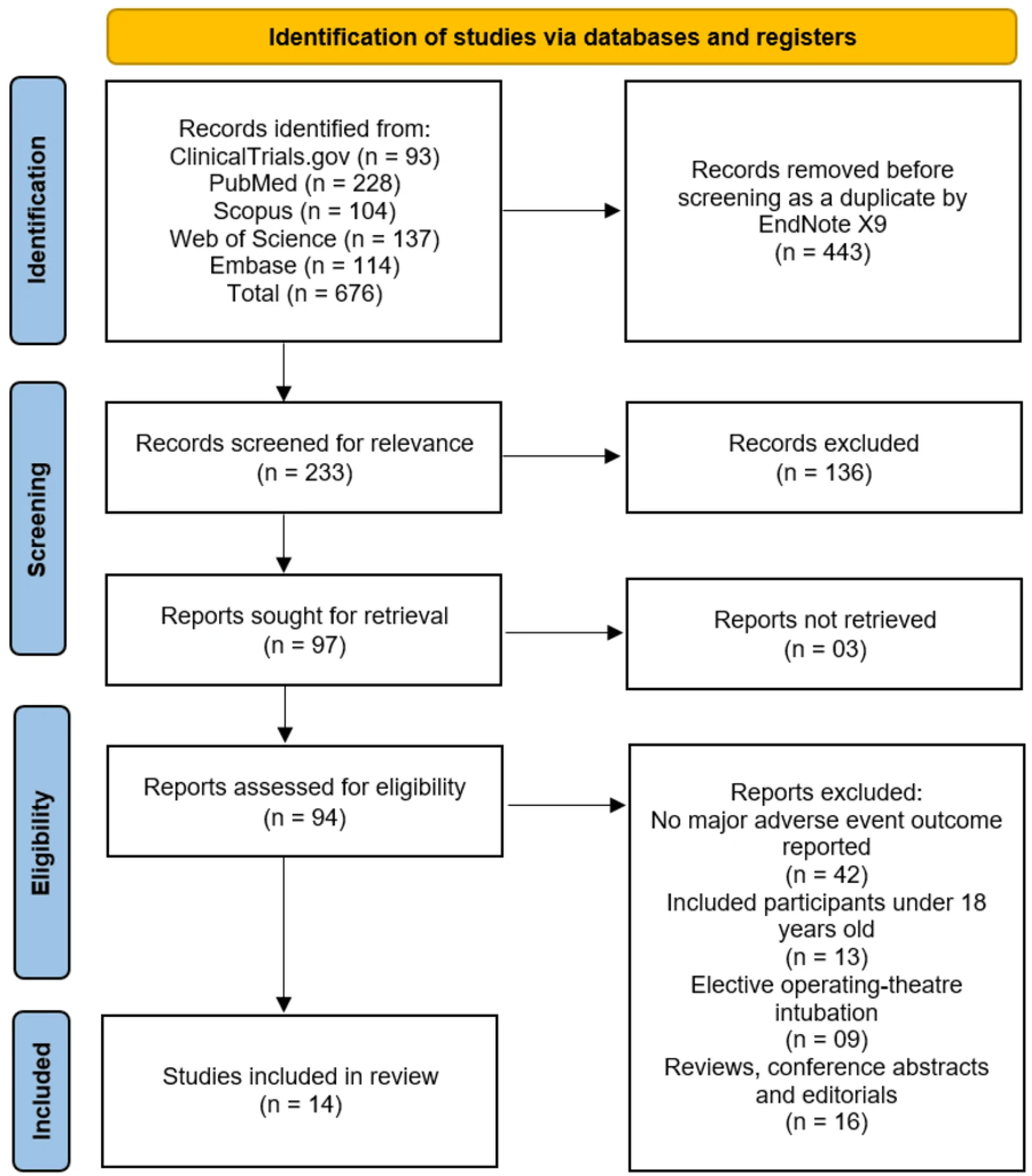
PRISMA 2020 flow diagram of study identification, screening, and inclusion PRISMA: Preferred Reporting Items for Systematic Reviews and Meta-Analyses

Database and registry searching identified 676 records: PubMed (n = 228), Web of Science (n = 137), Embase (n = 114), Scopus (n = 104), and ClinicalTrials.gov (n = 93). After the removal of 443 duplicates in EndNote X9, 233 records were screened for relevance, and 136 were excluded. Ninety-seven reports were sought for retrieval, of which three could not be obtained, leaving 94 reports assessed for eligibility in full text. Eighty reports were subsequently excluded: 42 reported no major adverse event outcome, 16 were reviews, conference abstracts, or editorials, 13 included participants under 18 years of age, and nine addressed elective operating-theatre intubation. Fourteen studies met all eligibility criteria and were included in the qualitative synthesis (Figure 1).

Characteristics of the Included Studies

The included studies were published between 2021 and 2026 and together comprised approximately 61,430 emergency intubation encounters (Table 2) [13-26]. Two were pragmatic multicentre randomised controlled trials [20,23], six were prospective cohort studies [14,17-19,24,25], three were retrospective or registry-based cohort analyses [13,15,22], two were cross-sectional observational studies [21,26], and one was a nested case-control study [16]. Geographically, five studies were conducted in the United States [13,18,20,22,23], two in Japan [19,25], and two in Ethiopia [15,26], one each was conducted in Taiwan [16], Pakistan [21], and Brazil [24], and two were international multicentre cohorts spanning 29 countries [14,17]. Sample sizes ranged from 112 [26] to 15,776 [13]. Nine studies were confined to the emergency department [13,15,16,18,19,21,22,24,25], two enrolled from emergency departments and intensive care units [20,23], two enrolled across emergency departments, intensive care units, and wards [14,17], and one included both emergency department and intensive care unit intubations [26]. Two reports [14,17] derived from the same international parent cohort and are treated as a single evidence source where their populations overlap; likewise, two registry analyses [13,22] drew on overlapping years of the same national airway registry.

**Table 2:** Characteristics of the 14 included studies aOR: adjusted odds ratio; AUC: area under the receiver operating characteristic curve; BARCO: Brazilian Airway Registry COoperation; CV: cardiovascular; DAPS: Difficult Airway Physiological Score; ED: emergency department; FPS: first-pass success; ICU: intensive care unit; IPTW: inverse probability of treatment weighting; JEAN: Japanese Emergency Airway Network; NEAR: National Emergency Airway Registry; OR: odds ratio; RSI: rapid sequence intubation; SBP: systolic blood pressure; INTUBE: International Observational Study to Understand the Impact and Best Practices of Airway Management in Critically Ill Patients; PREOXI: PRagmatic trial Examining OXygenation prior to Intubation; DEVICE: Direct versus Video Laryngoscope

Author, year (ref)	Country	Design	Population/setting	n	Intervention/exposure	Comparator	Outcomes	Key findings
April et al., 2021 [13]	USA	Registry cohort (NEAR)	Patients >14 y, ED	15,776	Pre-intubation physiology; device	No peri-intubation arrest	Peri-intubation cardiac arrest	Arrest 1.0%; SBP <100 mmHg aOR 6.2; SpO₂ <90% aOR 3.1
Russotto et al., 2021 [14]	29 countries	Prospective cohort (INTUBE)	Critically ill adults; ICU, ED, wards	2,964	Emergency intubation practice	None (descriptive)	Composite major adverse event; mortality	≥1 event 45.2%; CV instability 42.6%; hypoxaemia 9.3%; arrest 3.1%
Zewdie et al., 2021 [15]	Ethiopia	Retrospective cohort	All ED intubations, tertiary centre	256	RSI vs sedative-only vs no drugs	Technique subgroups	Success; immediate complications	FPS 70.3%; complications 28.5%; hypoxia commonest (n = 32)
Yang et al., 2022 [16]	Taiwan	Nested case-control	Adults, ED	5,130	Pre-intubation vitals, indication, labs	Matched patients without arrest	Peri-intubation cardiac arrest	Arrest 1.8%; pulmonary oedema aOR 5.92; SBP <90 mmHg aOR 5.22; lactate aOR 1.012
Russotto et al., 2022 [17]	29 countries	Prospective cohort (INTUBE secondary)	Critically ill adults with haemodynamic data	2,760	Induction agents; pre-induction fluids or vasopressors	No cardiovascular collapse	Cardiovascular collapse; ICU mortality	Collapse 43.4%; propofol OR 1.28 (IPTW 1.23); ICU death aOR 2.47
Mohr et al., 2023 [18]	USA	Prospective cohort (20 EDs)	Adults, ED, early pandemic	3,435	Known or suspected COVID-19	Non-COVID-19 patients	FPS; adverse events	FPS 87% vs 86%; adverse events 35% vs 19% (aOR 2.4)
Takahashi et al., 2023 [19]	Japan	Registry cohort (JEAN-2/5)	Adults ≥18 y, non-trauma, ED	7,349	Airway obstruction as indication	Non-obstructive indications	FPS; adverse events	FPS 74%, events 16% overall; obstruction: FPS aOR 0.60, events aOR 1.70
Prekker et al., 2023 [20]	USA	Randomised trial (DEVICE)	Critically ill adults, 17 EDs and ICUs	1,417	Video laryngoscope	Direct laryngoscope	First-attempt success; severe complications	FPS 85.1% vs 70.8%; severe complications 21.4% vs 20.9%
Waheed et al., 2024 [21]	Pakistan	Cross-sectional, prognostic model	Adults ≥18 y, ED, tertiary hospital	1,021	12 pre-intubation variables (DAPS)	No physiologically difficult airway	Composite serious outcome	Hypotension 379; arrest 124; DAPS AUC 0.857
April et al., 2024 [22]	USA	Registry cohort (NEAR)	Patients >14 y, ED	15,079	Number of intubation attempts	FPS	Major complications within 15 minutes	Any complication 14%; major 13%; OR 4.4, 7.4, 13.9, 9.3 by attempt
Gibbs et al., 2024 [23]	USA	Randomised trial (PREOXI)	Critically ill adults, 24 EDs and ICUs	1,301	Noninvasive ventilation preoxygenation	Oxygen mask	Hypoxaemia (SpO₂ <85%)	Hypoxaemia 9.1% vs 18.5%
Maia et al., 2025 [24]	Brazil	Prospective cohort, 18 EDs (BARCO)	Adults ≥18 y, ED	2,846	Emergency intubation	No major adverse event	Major adverse events; 28-day mortality	Events 32.3%; instability 20.0%, hypoxaemia 12.5%, arrest 3.5%; mortality 45.1%
Miwa et al., 2025 [25]	Japan	Prospective cohort (JEAN-5)	Adults, ED, 14 centres	1,984	COVID-19 at intubation	Non-COVID-19 patients	Adverse events; first-attempt success	Events 51.6% vs 17.1%; aOR 1.69; hypoxaemia commonest
Admas et al., 2026 [26]	Ethiopia	Prospective cross-sectional	Patients ≥13 y, ED and ICU, 2 hospitals	112	Direct laryngoscopy; operator grade	FPS vs failure	FPS; complications	FPS 64.3%; complications 37.5%; instability 22.3%; arrest 5.4%

Composite Major Adverse Event Burden

Composite major adverse event rates varied approximately fourfold across the included studies, largely reflecting differences in case mix and in event definitions (Table 3). The highest burden was reported in the international cohort of 2,964 critically ill patients from 197 sites in 29 countries, in which at least one major adverse event followed 45.2% of intubations [14]. In a national prospective cohort of 2,846 emergency department intubations in Brazil, major adverse events, defined as severe hypoxaemia, new haemodynamic instability, or cardiac arrest within 30 minutes, occurred in 919 patients (32.3%) [24]. A Japanese multicentre registry analysis reported adverse events in 51.6% of intubations of patients with COVID-19 compared with 17.1% of those without (adjusted odds ratio (aOR) 1.69; 95% confidence interval (CI): 1.17-2.42) [25], while a separate analysis of the same national network reported an overall adverse event rate of 16% among 7,349 patients [19]. Lower composite rates were reported in registry analyses applying narrower definitions: 14% of 15,079 emergency department encounters experienced any complication, with major complications in 13% [22]. Single-centre series from resource-limited settings reported intermediate rates of 28.5% among 256 intubations [15] and 37.5% among 112 intubations [26].

**Table 3:** Major adverse event profile and mortality across the included studies aOR: adjusted odds ratio; AUC: area under the receiver operating characteristic curve; DAPS: Difficult Airway Physiological Score; ED: emergency department; ICU: intensive care unit; NR: not reported; OR: odds ratio; SBP: systolic blood pressure

Author, year (ref)	Composite event rate	Cardiovascular instability	Severe hypoxaemia	Cardiac arrest	First-pass success	Mortality/other
April et al., 2021 [13]	NR	SBP <100 mmHg predicted arrest (aOR 6.2)	SpO₂ <90% predicted arrest (aOR 3.1)	1.0% (157/15,776)	NR	Arrest clustered in pre-intubation shock
Russotto et al., 2021 [14]	45.2%	42.6%	9.3%	3.1%	NR	ICU and 28-day mortality reported
Zewdie et al., 2021 [15]	28.5% (73/256)	Hypotension (n = 25)	Hypoxia (n = 32)	NR	70.3%; overall 99.2%	2 failed airways needed surgical rescue
Yang et al., 2022 [16]	NR	SBP 104 vs 136.5 mmHg in cases	NR	1.8% (92/5,130)	NR	Predictors: pulmonary oedema, SBP <90 mmHg, lactate
Russotto et al., 2022 [17]	NR	43.4% (1,199/2,760)	NR	Within collapse composite	NR	ICU mortality aOR 2.47
Mohr et al., 2023 [18]	35% vs 19%	NR	Hypoxia commonest event	NR	87% vs 86%	Adverse events aOR 2.4 with COVID-19
Takahashi et al., 2023 [19]	16%; 28% with obstruction	NR	NR	NR	74%; 63% with obstruction	Obstruction: FPS aOR 0.60; events aOR 1.70
Prekker et al., 2023 [20]	21.4% vs 20.9%	Within composite	Within composite	Within composite	85.1% vs 70.8%	Oesophageal intubation and aspiration similar
Waheed et al., 2024 [21]	Modelled by DAPS	Hypotension (n = 379)	SpO₂ <92% (n = 131)	124/1,021	NR	7 ED deaths; DAPS AUC 0.857
April et al., 2024 [22]	14% any; 13% major	Within major complications	Hypoxia commonest	Within major complications	13,459/15,079	OR rose with each attempt (4.4-13.9)
Gibbs et al., 2024 [23]	NR	NR	9.1% vs 18.5%	NR	NR	Preoxygenation halved hypoxaemia
Maia et al., 2025 [24]	32.3% (919/2,846)	20.0%	12.5%	3.5%	Reported	28-day mortality 45.1%
Miwa et al., 2025 [25]	51.6% vs 17.1%	NR	Hypoxaemia commonest	NR	Higher with COVID-19	Adverse events aOR 1.69
Admas et al., 2026 [26]	37.5% (42/112)	22.3%	2.7% during attempts	5.4%	64.3%	1-hour death 2.7%; tube misplacement 18.8%

Cardiovascular Instability and Collapse

Cardiovascular instability was the single most frequent major adverse event in every study that reported event-specific rates. In the international cohort, cardiovascular instability complicated 42.6% of intubations, far exceeding all other events [14]; a dedicated secondary analysis of that cohort identified peri-intubation cardiovascular instability or collapse in 1,199 of 2,760 patients (43.4%) [17]. New haemodynamic instability affected 20.0% of the Brazilian emergency department cohort [24], and cardiovascular instability affected 22.3% of the Ethiopian prospective cohort [26]. In a single-centre Pakistani cohort of 1,021 emergency department intubations, post-intubation hypotension was documented in 379 patients [21]. Independent correlates of cardiovascular collapse included older age (odds ratio (OR): 1.02 per year; 95% CI: 1.02-1.03), higher pre-induction heart rate, lower systolic blood pressure, lower oxygen saturation-to-inspired oxygen fraction ratio, and the use of propofol for induction (OR: 1.28; 95% CI: 1.05-1.57), the last of which remained the only factor independently associated with collapse after inverse probability of treatment weighting (OR: 1.23; 95% CI: 1.02-1.49) [17].

Severe Hypoxaemia

Severe hypoxaemia was the second most common major event and, in registry analyses using broader thresholds, the most frequently recorded complication overall. Severe hypoxaemia complicated 9.3% of intubations in the international cohort [14] and 12.5% in the Brazilian cohort [24], while hypoxia was the most common major complication in both national registry analyses [22,25] and the most common complication in the Ethiopian retrospective series, affecting 32 of 256 patients [15]. The magnitude of the problem and its modifiability were quantified by a multicentre randomised trial of preoxygenation strategy in 1,301 critically ill adults undergoing emergency intubation: hypoxaemia, defined as an oxygen saturation below 85% between induction and two minutes after intubation, occurred in 57 of 624 patients (9.1%) assigned to noninvasive ventilation compared with 118 of 637 patients (18.5%) assigned to an oxygen mask [23]. Notably, in the Ethiopian prospective cohort, hypoxaemia during attempts was recorded in only 2.7% of cases, an unexpectedly low figure that the investigators attributed in part to the absence of continuous waveform capnography and to reliance on operator self-report [26].

Peri-Intubation Cardiac Arrest

Peri-intubation cardiac arrest was the least frequent but most consequential major adverse event, with reported incidences spanning 1.0% to 5.4%. In the largest dedicated analysis, 157 of 15,776 emergency department intubations (1.0%; 95% CI: 0.9-1.2%) were complicated by peri-intubation cardiac arrest [13]. A nested case-control study of 5,130 eligible emergency department intubations identified 92 arrests (1.8%) [16]. Cardiac arrest complicated 3.1% of intubations in the international cohort [14], 3.5% in the Brazilian cohort [24], and 5.4% in the small Ethiopian prospective cohort, in which death within one hour occurred in 2.7% [26]. In the Pakistani cohort, cardiac arrest was documented in 124 of 1,021 patients [21]. The physiological antecedents of arrest were highly consistent across settings: pre-intubation systolic blood pressure below 100 mmHg (aOR: 6.2; 95% CI: 2.5-8.5) and pre-intubation oxygen saturation below 90% (aOR: 3.1; 95% CI: 2.0-4.8) predicted arrest in the registry analysis [13], while cardiogenic pulmonary oedema as the indication for intubation (aOR: 5.92; 95% CI: 1.04-33.57), systolic blood pressure below 90 mmHg (aOR: 5.22; 95% CI: 1.48-18.34), and elevated lactate (aOR: 1.012; 95% CI: 1.002-1.022) were independent predictors in the case-control study [16].

Determinants of Major Adverse Events

Procedural determinants of harm were dominated by the number of laryngoscopy attempts. In a registry analysis of 15,079 emergency department encounters, of which 13,459 were intubated on the first attempt, the adjusted odds of major complications relative to first-pass success rose to 4.4 (95% CI: 3.6-5.3) at the second attempt, 7.4 (95% CI: 5.0-10.7) at the third, 13.9 (95% CI: 5.6-34.3) at the fourth, and 9.3 (95% CI: 2.1-41.7) at the fifth or later attempt [22]. Anatomical and situational factors that reduce first-pass success were correspondingly associated with harm: intubation for airway obstruction reduced first-pass success (63% versus 74%; aOR: 0.60; 95% CI: 0.46-0.80) and nearly doubled the crude adverse event rate (28% versus 16%; aOR: 1.70; 95% CI: 1.27-2.29) [19]. Operator training level was the dominant modifiable predictor of first-pass success in the Ethiopian prospective cohort, where senior residents achieved markedly higher odds of single-pass intubation than first-year residents [26]. By contrast, device selection did not translate into fewer major events: although video laryngoscopy substantially improved first-pass success over direct laryngoscopy (85.1% versus 70.8%; absolute risk difference: 14.3 percentage points; 95% CI: 9.9-18.7), severe complications occurred with near-identical frequency in the two groups (21.4% versus 20.9%; absolute risk difference: 0.5 percentage points; 95% CI: -3.9 to 4.9) [20]. Patient-level physiological derangement also predicted harm independently of technique, and a 12-variable pre-intubation physiological score derived and internally validated in 1,021 emergency department intubations discriminated serious outcomes with an area under the receiver operating characteristic curve of 0.857 [21]. Finally, patient category modified risk: known or suspected COVID-19 was associated with a more than twofold increase in adjusted odds of adverse events among patients with low anticipated airway difficulty (35% versus 19%; aOR: 2.4; 95% CI: 1.7-3.3) despite equivalent first-pass success [18].

Association With Mortality

Three included studies linked major adverse events directly to survival. Patients experiencing peri-intubation cardiovascular instability or collapse had substantially higher intensive care unit mortality (aOR: 2.47; 95% CI: 1.72-3.55) [17]. In the Brazilian cohort, in which overall 28-day mortality was 45.1%, patients sustaining any major adverse event had higher mortality than those who did not [24]. In the Ethiopian prospective cohort, death within one hour of intubation occurred in 2.7% of cases and was concentrated among patients experiencing cardiovascular instability or arrest [26].

Risk of Bias

Methodological quality is summarised in Table 4 and Table 5. Of the 12 non-randomised studies, seven were rated low risk of bias (NOS 7-9) and five moderate risk (NOS 5-6); none was rated high risk. Recurrent limitations were reliance on operator self-report for adverse event ascertainment, incomplete adjustment for illness severity, and single-centre design. Both randomised trials were judged to be at low overall risk of bias on RoB 2, with the unavoidable absence of blinding of participants and personnel offset by objective, protocol-defined outcome measurement; the trial of laryngoscope type was stopped early for efficacy at a single preplanned interim analysis, which was pre-specified and does not alter the overall judgement.

**Table 4:** Newcastle-Ottawa Scale assessment of the 12 non-randomised studies

Author, year (ref)	Selection (max 4)	Comparability (max 2)	Outcome/exposure (max 3)	Total (max 9)	Risk of bias
April et al., 2021 [13]	4	2	3	9	Low
Russotto et al., 2021 [14]	4	2	3	9	Low
Zewdie et al., 2021 [15]	3	1	2	6	Moderate
Yang et al., 2022 [16]	4	2	2	8	Low
Russotto et al., 2022 [17]	4	2	3	9	Low
Mohr et al., 2023 [18]	4	2	3	9	Low
Takahashi et al., 2023 [19]	4	2	2	8	Low
Waheed et al., 2024 [21]	3	1	2	6	Moderate
April et al., 2024 [22]	4	2	3	9	Low
Maia et al., 2025 [24]	4	2	3	9	Low
Miwa et al., 2025 [25]	3	2	2	7	Low
Admas et al., 2026 [26]	3	1	1	5	Moderate

**Table 5:** Cochrane RoB 2 assessment of the two randomised controlled trials PREOXI: PRagmatic trial Examining OXygenation prior to Intubation; DEVICE: Direct versus Video Laryngoscope

Author, year (ref)	D1: Randomisation	D2: Deviations	D3: Missing data	D4: Outcome measurement	D5: Reported result	Overall
Prekker et al., 2023 (DEVICE) [20]	Low	Low	Low	Low	Low	Low
Gibbs et al., 2024 (PREOXI) [23]	Low	Low	Low	Low	Low	Low

Discussion

This systematic review of 14 contemporary primary studies, encompassing approximately 61,430 emergency intubation encounters across 14 countries, establishes three findings with direct implications for practice. First, major adverse events are not rare complications of emergency ETI but an expected feature of it, occurring in between roughly one in seven and one in two procedures depending on case mix and definitional threshold. Second, the hierarchy of events is remarkably stable across health systems: cardiovascular instability dominates, severe hypoxaemia follows, and cardiac arrest, though least frequent, is far commoner than in any elective airway population. Third, and most importantly for intervention design, the principal determinants of harm are physiological rather than anatomical, and procedural rather than technological, pre-existing shock, hypoxaemia, and acidaemia, compounded by each additional laryngoscopy attempt, account for far more of the observed variation in outcome than the choice of laryngoscope.

Variability in Event Rates

The fourfold variation in composite event rates across the included studies, from 14% in a national registry analysis [22] to 45.2% in an international critical care cohort [14], is at first sight difficult to reconcile. It is, however, largely explicable by two mechanisms. The first is case mix. Cohorts that enrolled critically ill patients irrespective of location, including those already receiving vasoactive support, reported the highest rates [14,17], whereas emergency department registries capturing all-comers, including patients intubated for isolated depressed consciousness with intact haemodynamics, reported the lowest [22]. The second mechanism is definitional. Studies that classified any new or increased vasopressor requirement as cardiovascular instability necessarily identified far more events than those requiring a systolic blood pressure below 65 mmHg. This is not a minor methodological quibble: the same patient may be counted as having sustained a major adverse event in one framework and none in another, which makes cross-study benchmarking hazardous and quantitative pooling of composite rates inadvisable. The consistency of the event hierarchy despite this definitional noise is therefore all the more striking and suggests that the underlying pathophysiology, namely, loss of sympathetic tone at induction, the transition from negative to positive intrathoracic pressure, and reduced venous return in a volume-depleted circulation, operates uniformly wherever emergency intubation is performed.

Cardiovascular Instability as the Dominant Event

Our finding that cardiovascular instability is the dominant event contextualises a body of work that had, until recently, focused disproportionately on oxygenation. A previous synthesis of critically ill populations similarly identified a high aggregate prevalence of peri-intubation major adverse events and noted wide between-study heterogeneity [4], and the present review extends that observation by demonstrating that the pattern holds in emergency department populations specifically and in low- and middle-income settings as well as high-income ones. The identification of propofol as the only induction agent independently associated with cardiovascular collapse after inverse probability weighting [17] is consistent with a study comparing ketamine and etomidate in critically ill adults, which examined the comparative haemodynamic profile of induction agents in this population [23]. Taken together, these data support a pragmatic conclusion that has been slow to reach the bedside: in the haemodynamically marginal patient, the choice and dose of induction agent is a first-order safety decision, not a matter of operator preference.

The Relationship Between Attempts and Harm

The relationship between attempts and harm deserves particular emphasis because it is both steep and modifiable. The near-14-fold adjusted odds of major complication at the fourth attempt relative to first-pass success [22] provides a quantitative foundation for the long-standing clinical maxim that the best attempt is the first attempt and aligns closely with the seminal single-centre observation that adverse events rise sharply once first-pass success is not achieved [1]. This finding also reframes the interpretation of device trials. The randomised comparison of video and direct laryngoscopy demonstrated a 14.3-percentage-point absolute improvement in first-pass success without any corresponding reduction in severe complications [20]. Superficially paradoxical, this result is best understood as evidence that the marginal intubations converted from failure to success by video laryngoscopy were not, for the most part, those in which physiological reserve was most precarious; the patients who arrest peri-intubation are largely those who arrive shocked, hypoxaemic, and acidotic, and a better view of the glottis does not correct any of those derangements. A comparable dissociation is visible in the observation that first-pass success was unaffected by known or suspected COVID-19 status while the adjusted odds of adverse events more than doubled [18] and again in the Japanese registry data, in which first-attempt success was higher in patients with COVID-19 even as their adverse event rate was threefold greater [25]. The implication is that improvements in first-pass success and reductions in major adverse events are related but non-identical goals and that quality improvement programmes measuring only the former will systematically underestimate residual harm.

Interventions Targeting Physiology

By contrast, interventions targeting physiology directly do appear to shift major outcomes. The randomised trial of preoxygenation with noninvasive ventilation halved the incidence of hypoxaemia during emergency intubation, from 18.5% to 9.1% [23], a treatment effect substantially larger than anything achieved by device substitution. Attempts to modify the haemodynamic axis have been less uniformly successful: a randomised trial of routine fluid bolus administration before intubation of critically ill adults did not reduce cardiovascular collapse [27], suggesting that indiscriminate volume loading is not the answer but directing attention instead toward vasopressor readiness, careful induction agent selection and dosing, and correction of the underlying shock state before laryngoscopy where the clinical situation permits any delay at all. The consistency with which pre-intubation hypotension, hyperlactataemia, acidaemia, and hypoxaemia predicted cardiac arrest across an American registry [13], a Taiwanese case-control study [16], and a Pakistani cohort [21] and the strong discrimination achieved by a pre-intubation physiological score in the last of these [21] collectively argue that risk in this domain is largely foreseeable [28]. This aligns with a systematic review and meta-analysis of the incidence and predisposing factors for peri-intubation cardiac arrest, which likewise located the principal risk in pre-procedural physiological derangement [29].

Mortality Association

The mortality signal identified in this review is important and should temper any tendency to regard peri-intubation events as transient physiological perturbations. Cardiovascular collapse conferred a nearly 2.5-fold increase in adjusted odds of intensive care unit death [17], and in a national emergency department cohort with 45.1% overall 28-day mortality, patients sustaining a major adverse event fared worse than those who did not [24]. Whether this association is causal or reflects confounding by illness severity cannot be resolved by observational data alone, and the included studies varied in the rigour of their adjustment. Nonetheless, the biological plausibility is strong, the direction of effect is consistent, and the effect sizes are large. From a clinical governance perspective, this is sufficient to justify treating major peri-intubation adverse events as patient safety incidents worthy of systematic capture and review, rather than as an accepted cost of a necessary procedure.

Resource-Limited Settings

The three studies conducted in resource-limited settings [15,21,26] deserve separate comment because they expose a global equity dimension that high-income registries cannot. First-pass success of 64.3% and 70.3% in the Ethiopian series [15,26] falls well below the approximately 84% pooled benchmark derived from predominantly high-income emergency department cohorts [2], and the accompanying complication rates of 37.5% and 28.5% are correspondingly high. The proximate causes identified by the investigators were the exclusive use of direct laryngoscopy, the absence of waveform capnography, and the predominance of junior operators [26]. Each of these is remediable at modest cost, and the observation that operator training year was the dominant independent predictor of first-pass success suggests that supervised training programmes may yield larger returns in such settings than equipment procurement alone. This inference is congruent with registry evidence from high-income systems showing stepwise improvement in first-pass success across postgraduate training years [7]. At the same time, the paradoxically low reported rate of intra-attempt hypoxaemia in one of these cohorts [26] illustrates how the absence of continuous monitoring can render adverse events invisible in exactly the settings where they are most likely to occur, a form of ascertainment bias that almost certainly causes the global burden of peri-intubation harm to be underestimated.

Knowledge Gaps

Several knowledge gaps follow directly from this synthesis. The most pressing is definitional. Until the emergency airway community converges on a standard core outcome set with explicit thresholds for hypoxaemia, hypotension, and the observation window, incidence estimates will remain incomparable, and meta-analysis of composite outcomes will remain uninterpretable. Second, almost all of the determinants identified here derive from observational adjustment, and few have been subjected to randomised evaluation; the contrast between the substantial benefit of a randomised preoxygenation strategy [23] and the null result of a randomised fluid strategy [27] shows that intuitively attractive interventions require testing rather than assumption. Third, peri-intubation risk prediction remains immature: the one score identified in this review was derived and validated within a single centre [21] and awaits external validation, and no included study evaluated whether prospective use of a risk score changes management or outcome. Fourth, the evidence base is geographically skewed, with no included study from South Asia outside a single Pakistani centre, none from West Africa, and none from Latin America apart from one Brazilian cohort [24]. Fifth, outcomes beyond the immediate peri-intubation window, namely, neurological function, ventilator-free days, and functional recovery, were almost entirely unreported, so the downstream cost of a peri-intubation event remains largely unquantified. Finally, current guidance on the difficult airway remains oriented predominantly toward anatomical difficulty [3], and the translation of physiological risk stratification into actionable algorithmic guidance is incomplete.

Implications for Practice

For practice, the evidence assembled here supports a coherent set of priorities. Every emergency intubation should be preceded by the explicit assessment of physiological risk, with particular attention to systolic blood pressure, shock index, oxygen saturation, and acid-base status, since these variables predict the events that kill patients. Preoxygenation should be optimised, and noninvasive ventilation should be strongly considered in hypoxaemic patients on the strength of randomised evidence [23]. Vasopressors should be immediately available and induction agents chosen and dosed with the circulation in mind. The procedure should be configured to maximise the probability of success on the first attempt, most experienced available operator, optimal positioning, a bougie or stylet readily to hand, and video laryngoscopy where available, while recognising that first-pass success is a means to reducing harm rather than the endpoint itself. Departments should audit major adverse events alongside first-pass success, using pre-specified definitions, because the two metrics can move independently.

Limitations

Limitations of the Included Studies

This review has several limitations relating to the primary evidence. Substantial clinical and methodological heterogeneity across the included studies precluded quantitative pooling, so all findings are synthesised narratively and no summary incidence estimate is offered. Definitions of major adverse events differed materially between studies, particularly the thresholds applied to hypoxaemia and hypotension and the duration of the post-intubation observation window, which limits the comparability of the reported incidences. The evidence base is dominated by observational designs in which residual confounding by illness severity cannot be excluded, and adverse event ascertainment relied on operator self-report in most cohorts, a method likely to under-detect events. Two pairs of included reports drew on overlapping parent cohorts, so the total number of encounters reported here is not a count of wholly independent patients.

Limitations of the Review Process

The review process itself has constraints. The protocol was not prospectively registered in an international registry. A five-year date restriction was applied a priori to ensure contemporary relevance, which necessarily excludes older studies that contributed foundational evidence, and the reference limit imposed by the target journal constrained the breadth of contextual citation. Study authors were not contacted for unreported data, and the absence of a meta-analysis meant that publication bias could not be formally assessed, although the predominance of large registry and multicentre cohort studies makes selective non-publication of null findings a less likely source of distortion in this literature than in smaller trial-based fields.

## Conclusions

Major adverse events complicate a substantial proportion of emergency ETI in adults, with reported composite incidences ranging from approximately 14% to 52% across contemporary studies. Cardiovascular instability is consistently the dominant event, followed by severe hypoxaemia and peri-intubation cardiac arrest, and these events are independently associated with mortality. The principal determinants of harm are pre-intubation physiological derangement, hypotension, hypoxaemia, acidaemia, and hyperlactataemia, together with the number of laryngoscopy attempts required. Interventions that address physiology, such as optimised preoxygenation, reduce major adverse events, whereas interventions that improve first-pass success alone may not. Emergency airway programmes should therefore incorporate structured pre-intubation physiological risk assessment, haemodynamic preparation, and routine audit of major adverse events using standardised definitions, in addition to established first-pass success metrics.

## Appendices

**Table 6:** Search strings for databases

Database	Search string
PubMed/MEDLINE	("intubation, intratracheal"[MeSH] OR "endotracheal intubation" OR "tracheal intubation" OR "airway management" OR "rapid sequence intubation" OR "laryngoscopy") AND ("emergency" OR "emergency department" OR "emergency service, hospital" OR "critically ill" OR "resuscitation" OR "non-elective") AND ("adverse event" OR "complication" OR "peri-intubation" OR "hypoxaemia" OR "hypoxemia" OR "desaturation" OR "hypotension" OR "cardiovascular collapse" OR "haemodynamic instability" OR "cardiac arrest" OR "oesophageal intubation" OR "mortality")
Embase	('endotracheal intubation'/exp OR 'tracheal intubation' OR 'airway management' OR 'rapid sequence intubation' OR 'laryngoscopy') AND ('emergency' OR 'emergency department' OR 'emergency ward' OR 'critically ill patient' OR 'resuscitation' OR 'non-elective') AND ('adverse event'/exp OR 'complication'/exp OR 'peri-intubation' OR 'hypoxemia' OR 'desaturation' OR 'hypotension' OR 'cardiovascular collapse' OR 'hemodynamic instability' OR 'heart arrest'/exp OR 'esophageal intubation' OR 'mortality'/exp)
Scopus	TITLE-ABS-KEY("endotracheal intubation" OR "tracheal intubation" OR "airway management" OR "rapid sequence intubation" OR "laryngoscopy") AND TITLE-ABS-KEY("emergency" OR "emergency department" OR "critically ill" OR "resuscitation" OR "non-elective") AND TITLE-ABS-KEY("adverse event" OR "complication" OR "peri-intubation" OR "hypoxaemia" OR "hypoxemia" OR "desaturation" OR "hypotension" OR "cardiovascular collapse" OR "haemodynamic instability" OR "cardiac arrest" OR "oesophageal intubation" OR "mortality")
Web of Science	TS=("endotracheal intubation" OR "tracheal intubation" OR "airway management" OR "rapid sequence intubation" OR "laryngoscopy") AND TS=("emergency" OR "emergency department" OR "critically ill" OR "resuscitation" OR "non-elective") AND TS=("adverse event" OR "complication" OR "peri-intubation" OR "hypoxaemia" OR "hypoxemia" OR "desaturation" OR "hypotension" OR "cardiovascular collapse" OR "haemodynamic instability" OR "cardiac arrest" OR "oesophageal intubation" OR "mortality")
ClinicalTrials.gov	(intubation OR endotracheal intubation OR tracheal intubation OR airway management) AND (emergency OR critically ill) AND (adverse event OR complication OR hypoxemia OR hypotension OR cardiac arrest OR mortality)
